# Ovariectomy, but not moderate intermittent hypoxia, promotes respiratory instability and risk of obesity in adult female rats

**DOI:** 10.1113/EP094077

**Published:** 2026-09-24

**Authors:** Marianne Gagnon, Stéphanie Fournier, François Marcouiller, Natalie J. Michael, Vincent Joseph, Richard Kinkead

**Affiliations:** ^1^ Research Center of the Quebec Heart and Lung Institute Quebec City Quebec Canada; ^2^ Faculty of Pharmacy Université Laval Quebec City Quebec Canada; ^3^ Department of Pediatrics, Faculty of Medicine Université Laval Quebec City Quebec Canada; ^4^ Groupe de recherche en santé respiratoire (GESER) Université Laval Quebec City Quebec Canada

**Keywords:** HPA axis, intermittent hypoxia, leptin, menopause, ovarian hormones, respiratory control, sex‐based differences, sleep apnoea

## Abstract

Sleep apnoea (SA) is a prevalent and complex respiratory disorder associated with recurrent interruptions of air flow during sleep. SA causes sleep fragmentation and intermittent hypoxia which leads to numerous negative health consequences. While SA is typically more common in men, its prevalence in women increases sharply after menopause, implicating the loss of ovarian hormones in its pathogenesis. Despite the potential of hormone replacement therapy to modulate SA, the physiological mechanisms linking loss of ovarian function to SA remain unclear. Using an integrative approach, we investigated the combined effects of surgical removal of the ovaries (ovariectomy; OVX) and a 7‐day moderate intermittent hypoxia protocol (IH; 30s at $F_{iO_{2}}$ 0.10, 10 cycles/h, 8 h/day) on respiratory, metabolic, cardiovascular and neuroendocrine regulation, including hypothalamic–pituitary–adrenal (HPA) axis activation and leptin levels in adult female rats. OVX increased apnoea frequency, respiratory instability and ventilatory responses to O_2_ (10%, 120 s) but not CO_2_ (5%, 10 min). However, contrary to our hypothesis, moderate IH did not worsen metabolic or cardiorespiratory dysfunction caused by OVX. OVX did not affect mean arterial pressure or heart rate but augmented weight gain and activated the HPA axis. Thus, our results indicate that OVX independently promotes apnoeas and activation of the stress axis and this is not worsened by additional exposure to moderate IH. This underscores the predominant role of ovarian hormone deficiency in SA‐related pathophysiologies, highlighting menopause as a critical window for vulnerability to respiratory problems.

## Abstract (highlights)

- **What is the central question of this study?**
  In women, sleep apnoea (SA) and cardiometabolic comorbidities increase after menopause: does intermittent hypoxia (IH) exacerbate the physiological consequences of ovariectomy (OVX) in adult female rats?
- **What is the main finding and its importance?**
  OVX increased apnoeas and respiratory instability during sleep, promoted weight gain and activated stress responses. IH had no additional effects, underscoring the predominant role of ovarian hormone deficiency in SA pathophysiology and identifying menopause as a critical period of respiratory and metabolic vulnerability.

## 1 INTRODUCTION

Sleep apnoea (SA) is a highly prevalent respiratory disorder affecting nearly 1 billion adults worldwide (Benjafield et al., 2019). SA is characterized by recurrent apnoeic events that fragment sleep and are often accompanied by oxygen desaturation which stimulates the carotid bodies (American Academy of Sleep Medicine, 1999; Dempsey et al., 2010; Gastaut et al., 1966; Iturriaga et al., 2021). Repeated nocturnal episodes of apnoeas lead to chronic intermittent hypoxia, currently viewed as a major pathological mechanism driving SA‐related comorbidities including oxidative stress, metabolic disturbance and hypertension (Drager et al., 2010; Foster et al., 2007; Laouafa et al., 2019). While the prevalence of SA in men is well recognized, SA is generally overlooked as a health issue in women.

Sex‐based differences in SA manifestations are becoming increasingly recognized. Compared to men, women suffering from SA more frequently report morning headaches, depression, anxiety and insomnia (Baldwin et al., 2004; Basoglu & Tasbakan, 2018; Wahner‐Roedler et al., 2007; Ye et al., 2009). Despite SA being approximately 2.5 times more prevalent in men (Heinzer et al., 2015; Peppard et al., 2013), it disproportionately affects women after menopause. After menopause, the sex gap in prevalence narrows, with nearly half (47%) of postmenopausal women suffering from moderate to severe SA (Lin et al., 2008; Mirer et al., 2017; Valipour, 2012), emphasizing the role of hormonal changes in disease pathogenesis (Bixler et al., 2001; Sunwoo et al., 2018; Young et al., 2003). These data emphasize the importance of menopause as a turning point in disease onset and challenge the long‐standing perception of SA as a disorder mainly affecting men. We must keep in mind, however, that menopause is a natural physiological process, and the fact that not all ageing women develop SA suggests that these patients represent a distinct population with distinct physiological and health outcomes.

Despite its clinical manifestation, the reasons explaining such differences in the ageing trajectories of women are not fully understood, (Huang et al., 2018; Young et al., 2003). However, our recent studies suggest that age‐related loss in ovarian function reveals the latent effects of early life stress on cardiorespiratory and metabolic function in female rats (Ambrozio‐Marques et al., 2025, 2026; Fournier et al., 2015). In line with this concept, a growing body of evidence implicates menopause‐associated neuroendocrine changes, including altered hypothalamic–pituitary–adrenal (HPA) axis activity, in the increased vulnerability of postmenopausal women to respiratory and metabolic disturbances; however, its role in SA progression remains unclear (Woods et al., 2006). Given that chronic intermittent hypoxia is a potent activator of the HPA axis in male rats that can lead to sustained elevations in circulating corticosterone (Ma et al., 2008; Zoccal et al., 2007), this neuroendocrine interplay may contribute to disease exacerbation in postmenopausal women; however, its potential implication remains untested in females.

To address this issue, we investigated the hypothesis that exposure to 7 days of moderate intermittent hypoxia (IH) exacerbates the metabolic, cardiorespiratory and endocrine consequences resulting from the loss of ovarian function in adult female rats. In this study, we used ovariectomy (OVX) as an experimental model of age‑related reduction in ovarian hormones, and then explored the consequences of normoxia or IH. The aetiology of SA is complex and multifactorial, but our investigation focused on two important factors that increase the risk of SA, namely, the intensity of respiratory chemoreflexes (loop gain) and obesity. By integrating systemic and endocrine analyses, this study aimed to provide new insights into the mechanistic link between loss of ovarian function, including potential involvement of the stress system, and SA progression in women.

## 2 METHODS

### 2.1 Ethical approval

All protocols were conducted in accordance with guidelines outlined by the Canadian Council on Animal Care and approved by the Laval University Animal Care Committee (protocol no. 2020–441). All animal experimentation was in line with the ARRIVE 2.0 guidelines and the guidelines of the Canadian Council on Animal Care. All personnel involved in this research followed appropriate training and complied with the institutional and national ethical guidelines.

All animals were born and raised in the animal facilities of the Research Centre of the Quebec Heart and Lung Institute (Quebec City, Canada) under standard laboratory conditions with a 12‐h light–dark cycle (lights on at 07.00 h) and controlled temperature (22 ± 1°C) and humidity (55%). Food and water were provided ad libitum throughout the duration of the study. Adult male and nulliparous female Sprague–Dawley rat breeding pairs were obtained from Charles River Laboratories (Montréal, Canada) for the generation of all experimental animals. After confirmation of mating, pregnant females were individually housed and monitored daily until they gave birth to their litters (approximately 12–15 pups). To reduce variability due to litter size and sex composition, litters were standardized within 48 h of birth to 12 pups per dam, with balanced male‐to‐female ratios. Experiments were carried out on 34 of these female rats once they reached sexual maturity (8 weeks old, 200–230 g). At the end of the experiments, rats were deeply anaesthetized by a terminal intraperitoneal injection of a mixture of ketamine (80 mg kg^−1^; Pfizer, Kirkland, QC, Canada) and xylazine (10 mg kg^−1^, Bimeda, Cambridge, ON, Canada). Once a surgical plane of anaesthesia was confirmed by the absence of pedal withdrawal reflex, animals were transcardially perfused, and death was confirmed before tissue collection.

### 2.2 Experimental groups and procedures

**FIGURE 1:**
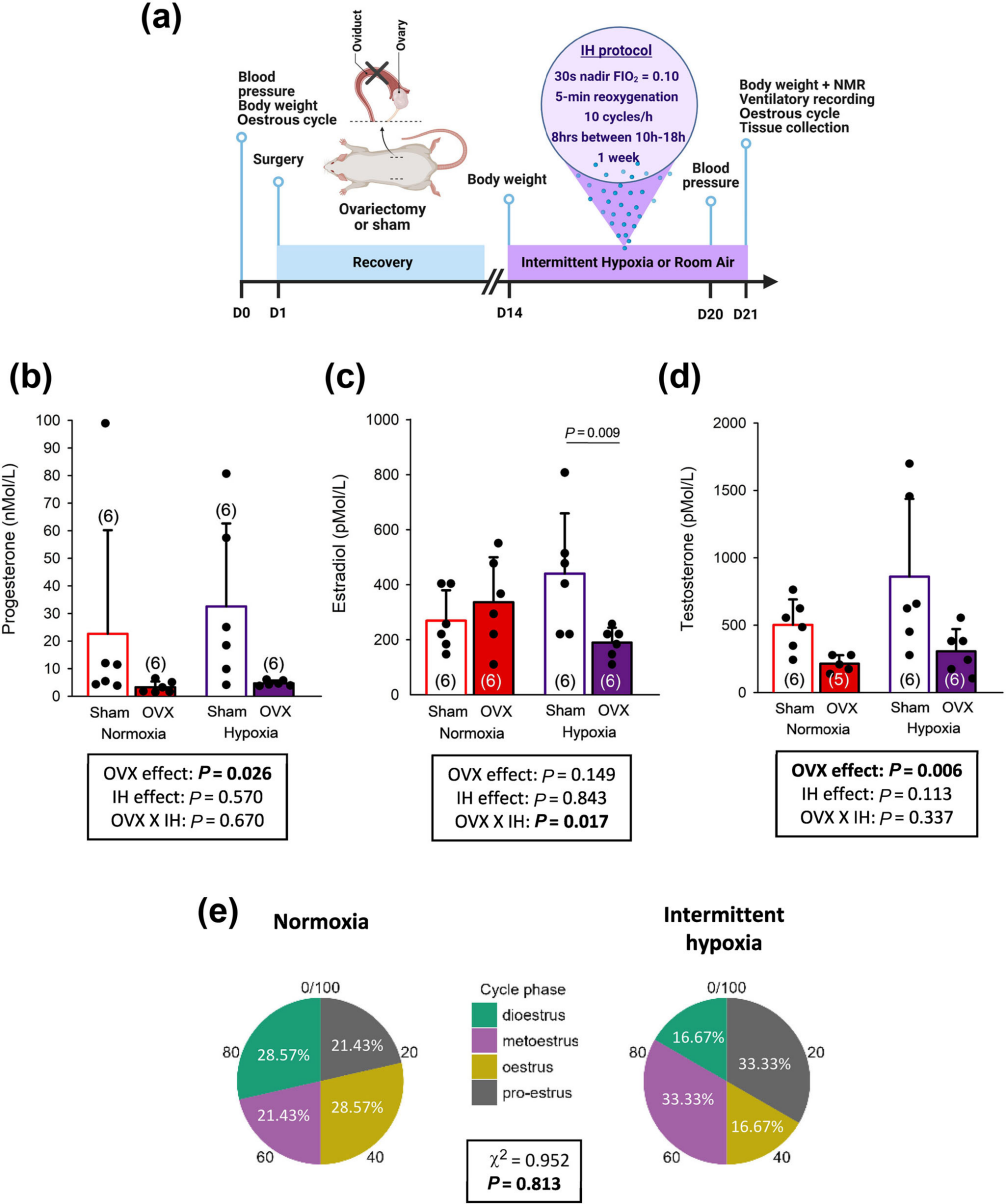
Validation of ovariectomy (OVX) and effects of intermittent hypoxia (IH) on sex‐steroid profile and oestrous cycle. (a) Representation of the experimental design and the times at which the OVX/sham surgery and physiological measurements were performed. Created in BioRender. Gagnon, M. (2026) https://BioRender.com/2wzxfxs. (b–d) Serum progesterone (b), oestradiol (c), and testosterone (d) levels analysed by a multiplex assay of female adult rat sham‐operated (open bars) or ovariectomized (OVX; filled bars) and exposed to normoxia (red bars) or intermittent hypoxia (IH; purple bars). Each rat is represented by an individual data point; bar height indicates group means ± SD. Group sample size (*n*) is indicated in parentheses for each group, and exact Bonferroni‐adjusted *post hoc P*‐values are shown directly on the figure when applicable. (e) Distribution of oestrous cycle phases in sham‐operated female rats exposed to normoxia or intermittent hypoxia. Pie charts show the relative proportion of animals in dioestrus, metoestrus, oestrus and pro‐oestrus in each condition.

We used four groups of female rats, all of which underwent surgical procedures of either ovariectomy (OVX) or sham and were exposed to either room air (normoxia) or intermittent hypoxia (IH), as described below. Figure 1a provides a schematic overview of the protocol. All measurements were performed between 08.00 h and 12.00 h to minimize variability related to the circadian rhythm.

#### 2.2.1 Ovariectomy

Rats underwent bilateral OVX or sham surgery according to standard procedures used in our laboratory (Fournier et al., 2025; Marques et al., 2021). Briefly, rats were anaesthetized with isoflurane in an induction chamber (3.5% in oxygen, 1.5 L/min) and then maintained on isoflurane delivered via a nose cone (2.5% in oxygen, 0.5 L/min) during the surgery. The depth of anaesthesia was verified by the absence of a pedal withdrawal reflex in response to hind‐paw pinch and monitored throughout the procedure. To minimize pain and distress, animals were placed on a heating pad, received ophthalmic ointment to prevent corneal drying, and were given warmed lactated Ringer's solution subcutaneously for perioperative fluid support. Animals received subcutaneous injections of local and systemic analgesics (lidocaine/bupivacaine, 0.25 mL; buprenorphine, 0.067 mL/100 g, respectively). The ovaries were accessed through a dorsal incision through the skin 1.5 cm below the ribs, and incision of the muscle layers on each flank. Rats assigned to the OVX group had both ovaries removed; each ovary was visualized, exteriorized with the associated periovarian adipose tissue, clamped and excised bilaterally. In sham‐operated rats, the same bilateral incisions were performed and the ovaries and associated tissues were visualized and manipulated in the same manner, but the ovarian tissue was neither clamped nor removed. The incision was closed by suturing the muscles and stapling the skin. All animals received postoperative meloxicam 0.1 mL/100 g by subcutaneous injection and were placed in a clean cage partially placed over a heating pad, provided with moist food, and monitored until awakening. Two‐weeks of recovery was provided before the initiation of the IH protocol to ensure washout of ovarian hormones (Figure 1a).

#### 2.2.2 Moderate intermittent hypoxia protocol

Animals were randomly assigned to either room air (control) or to a 7‐day IH protocol. The ventilated cages that house the animals were connected to an oxycycler (Biospherix, Redfield, NY, USA). Within their cage (internal volume 0.05 m^3^), rats intermittently received pure nitrogen, followed by room air for reoxygenation. Each bout of hypoxia lasted 30 s, reached a nadir $F_{iO_{2}}$ of 0.10 and was followed by 5 min of reoxygenation. Rats were exposed to 10 cycles per hour, for 8 h between 10.00 h and 18.00 h. This protocol is based on the original study of Fletcher et al. (1992) and aims to replicate the repeated de‐saturations and reoxygenation seen in human with SA. This protocol was chosen because a 1‐week protocol is sufficient to increase mean arterial blood pressure, especially in males and OVX females (Hinojosa‐Laborde & Mifflin, 2005). Moreover, this moderate depth of hypoxia is more clinically applicable compared to more severe protocols (Dematteis et al., 2009; Toth & Bhargava, 2013). Due to limited availability of the IH chambers, control rats remained in the normoxic housing room; they were not exposed to the intermittent hypoxia‐associated noise.

### 2.3 Animal monitoring and experimental measurements

#### 2.3.1 Body composition, oestrous cycle and cardiovascular measurements

Body weight, oestrous cycle phase and cardiovascular parameters were assessed at three distinct times over the course of the protocol: before surgery (day 0), before the IH protocol (day 14) and at the end of the experiments (day 21; Figure 1a). The oestrous cycle was determined by a vaginal smear after the completion of cardiovascular measurements using an established approach in our laboratory (Dumont et al., 2011; Fournier et al., 2025; Marques et al., 2021). For cardiovascular measurement, the animals were acclimatized to the cylindrical acrylic support and warming mat for 1 h prior to the experiment (‘tail cuff method’; CODA system; Kent Scientific, Torrington, CT, USA) (Laouafa et al., 2017). Unlike Laouafa et al. (2017), however, the blood pressure and heart rate values reported here reflect the mean of 10 consecutive data points, not just the three lowest values. We chose this approach because we considered it more representative of resting cardiovascular values in our experimental conditions. To verify whether the analytical approach influenced the interpretation, blood pressure data were also analysed using the three lowest values; this sensitivity analysis led to the same conclusion. As standard in our animal facility, animals were housed in pairs throughout the protocol. Food intake was measured per cage (food provided at day 0 minus food remaining at day 21), and the resulting value was divided by two to estimate average food intake per rat. Because OVX and sham rats were housed together, differences in food intake could not be determined between OVX and sham rats. Therefore, analysis of food intake is only presented by exposure condition (IH vs. normoxia). Body composition (fat, fluid and lean mass) was evaluated using quantitative nuclear magnetic resonance (NMR) technology after the IH protocol, on day 21 (Minispec LF90, Bruker Corporation, Billerica, MA, USA). Ventilatory measurements were performed after the IH protocol (day 21; details below).

#### 2.3.2 Ventilatory measurements

Ventilatory measurements were performed using a whole‐body flow‐through plethysmography system (EMKA technologies, Paris, France) according to standard laboratory procedures (Fournier et al., 2015; Laouafa et al., 2017). Briefly, the rats were placed in a 4.5‐L Plexiglas plethysmography chamber in which they could move freely. The airflow through the chamber was maintained between 1.5 and 2.0 L/min (VENT2; EMKA Technologies). Rats were acclimatized to the chamber for 2 h before experiments began by recording respiratory activity at rest (normoxia). All respiratory signals were recorded using Spike2 data acquisition software (version 7.20, Micro 1401 data acquisition system; Cambridge Electronic Design, Cambridge, UK). Water vapor pressure, O_2_ (Oxzilla FC‐2; Sable Systems International, North Las Vegas, NV, USA) and CO_2_ (CA‐10; Sable Systems International) levels were continuously measured using gas analysers. Barometric pressure was measured at the beginning and end of each plethysmography recording, whereas chamber temperature was monitored continuously. Composition of the gas mixtures flowing in and out of the chamber was used for subsequent calculation of oxygen consumption (${\dot{V}}_{O_{2}}$) and CO_2_ production (${\dot{V}}_{CO_{2}}$) in an open system (Mortola & Dotta, 1992), as described below. Rectal temperature was measured before and after the plethysmography recording and reported as the average of the two measures. Ventilatory measurements were performed between 09.00 h and 12.00 h, when rats normally sleep.

#### 2.3.3 Assessment of respiratory reflexes

Following 2 h of baseline recording, acute responses to respiratory stimuli were measured. The order of the stimuli was determined randomly. Hypoxia was induced by changing the incoming gas to pure nitrogen for 120 s. A nadir of 10% O_2_ was reached within 140 s, $F_{iO_{2}}$ then progressively returned to 0.21 within 10 min. The rats recovered for 1 h before being exposed to the next stimulus. Hypercapnia was induced by introducing a gas mixture of 21% O_2_ and 5% CO_2_ for 10 min. This was followed by a period of 1 h to return to normal with ambient air (normoxia).

#### 2.3.4 Respiratory signal analysis

Basal respiratory rate (*f*_R_), tidal volume (*V*_T_) and minute ventilation (${\dot{V}}_{E}$) values were obtained during non‐REM sleep. Non‐REM sleep was identified based on the on the calm behaviour and stability of the respiratory signals as previously described and validated by Bastianini et al. (2017). During data analysis, the software parameters were adjusted to eliminate non‐respiratory signals related to animal movement or sniffing. Body temperature, barometric pressure, room temperature and humidity were measured to express *V*_T_ in millilitres per 100 g according to the equations of Drorbaugh & Fenn (1955). ${\dot{V}}_{E}$ and ${\dot{V}}_{O_{2}}$ were also corrected for body weight and expressed in ml BTPS and STPD, respectively.

**FIGURE 2:**
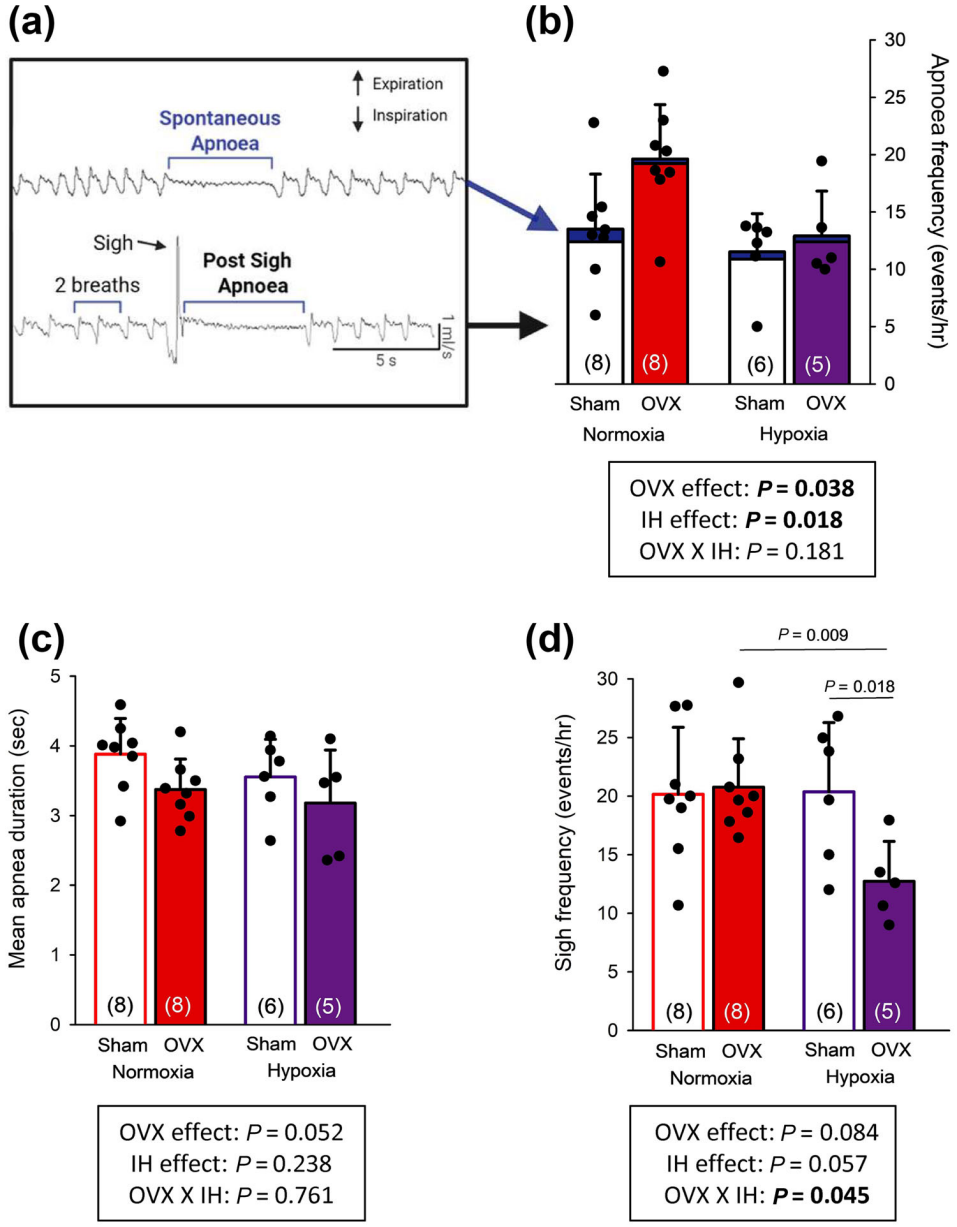
Effects of ovariectomy (OVX) and moderate intermittent hypoxia (IH) on apnoeic events during non‐REM sleep. (a) Representative whole‐body plethysmography recordings obtained in unrestrained rats during non‐REM sleep illustrating spontaneous apnoea (top trace) and post‐sigh apnoea (lower trace). Created in BioRender. Gagnon, M. (2026) https://BioRender.com/qw21yww. (b) Bar graph with individual data comparing the mean frequency of the total number of apnoeic events. The dark part (top) of the bar indicates the mean number of spontaneous apnoeas and the rest indicate post‐sigh events. Data are reported for female rats that were either sham‐operated (open bars) or ovariectomized (OVX; filled bars). Each group was exposed to normoxia (red bars) or intermittent hypoxia (purple bars). (c, d) Mean duration of post‐sigh apnoeas (c) and frequency of sighs (d) measured during non‐REM sleep. Each rat is represented by an individual data point; bar height indicates group means ± SD. Group sample size (*n*) is indicated in parentheses for each group, and exact Bonferroni‐adjusted *post hoc P*‐values are shown directly on the figure when applicable.

Apnoeas were defined using the criteria commonly used in our group (Gagnon et al., 2023; Ganouna‐Cohen et al., 2023). Briefly, apnoeas consist of respiratory pauses exceeding two typical respiratory cycles, determined in the same animal from a nearby stable baseline breathing period, after a sigh (post‐sigh apnoea) or without a preceding sigh (spontaneous apnoeas). Sighs were identified by inspiratory volumes at least double the normal tidal volume and followed by a rapid expiration (Figure 2a). Apnoeas were counted manually and expressed as the number of events per hour. The apnoea index consists of the total number of events (post‐sigh and spontaneous apnoeas) expressed per hour of non‐REM sleep. The duration of each apnoeic event was measured from the onset of the apnoea to the return of respiratory activity.

Analysis of the hypoxic ventilatory response focused on the rapid increase of breathing frequency at the onset of hypoxia; this acute phase reflects carotid body activation (Powell et al., 1998). The peak frequency increase measured at 2 min was expressed as a percentage change from baseline. Because the ventilatory response to hypercapnia typically reaches ‘steady state’ within 5 min and involves tidal volume, we measured ${\dot{V}}_{E}$ during the last 5 min of CO_2_ exposure; the rat's response was then expressed as a percentage change from baseline.

#### 2.3.5 Tissue sampling and hormone analyses

Once plethysmography measurements were completed, rats recovered for 1 h in ambient air. Animals were then deeply anaesthetized with ketamine/xylazine as described previously. Terminal blood samples were obtained from the left ventricle by intracardiac puncture; they were then perfused intracardially with a phosphate‐buffered saline (PBS) solution (1 mL/g body weight, pH 7.4), followed by 4% paraformaldehyde (PFA) in PBS at the same volume ratio (1 mL/g). After perfusion, brains were carefully extracted, post‐fixed in 4% PFA at 4°C for 48 h and then transferred to a 20% sucrose solution for cryoprotection, also at 4°C for 48 h. Following this process, brains were frozen on dry ice and stored at −80°C until sectioning. Blood was placed in a serum‐gel and plasma‐gel clotting activator microtube (Sarstedt, Nümbrecht, Germany) and separated by centrifugation at 10,000 *g* for 10 min at 4°C and stored at −80°C until assayed.

Serum oestradiol, progesterone, testosterone (Millipore MSHMAG‐21K) and plasma leptin (Millipore RECYTMAG‐65K) were measured by a bead‐based multiplex assay, following the manufacturer's instructions (EMD Millipore, Billerica, MA, USA). Bead complexes were read on a Run plate on Luminex 200™ and analysed by MAGPIX with xPONENT software. Plasma adrenocorticotropic hormone (ACTH) and corticosterone levels were determined by Eve Technologies (Calgary, AB, Canada) using a multiplex assay (Millipore RSHMAG‐69K, EMD Millipore).

#### 2.3.6 FosB immunohistochemistry

FosB is a transcription factor commonly used as a marker of sustained neuronal activation (Nestler et al., 2001). The expression of FosB is slower (peaks after 6 h) than other commonly used immediate early genes (e.g., cFos which peaks after 2 h). Consequently, an increase in FosB expression is generally associated with chronic stress rather than an acute response to the experimental protocol. Immunolabeling was therefore conducted in tissue sections containing the nucleus tractus solitarius (NTS), the primary projection site of the carotid bodies (Finley & Katz, 1992), and as it also projects to the retrotrapezoid nucleus, a key site in central CO_2_ chemoreception (Guyenet et al., 2010). We also evaluated FosB expression in the paraventricular nucleus of the hypothalamus (PVN) as it regulates the stress response, blood pressure and respiratory reflexes (Ruyle et al., 2023; Tenorio‐Lopes & Kinkead, 2021). Moreover, the PVN also has direct projections to the NTS (Ruyle et al., 2019). Tissue sections containing these structures were identified, and immunohistochemistry was performed using an established free‐floating protocol (Ambrozio‐Marques et al., 2026; Ansorg et al., 2015). Briefly, frozen brains were cut into 40 µm coronal slices using a sliding microtome with the ‘dry ice’ method (Anders, 1942). These sections were then placed in a cold cryoprotectant solution consisting of 0.05 M sodium phosphate buffer, 30% ethylene glycol and 20% glycerol, and stored at −20°C. Free‐floating tissue sections were initially rinsed in Tris‐buffered saline (TBS), pH 7.4, for 1 min at room temperature. Throughout the procedure, tissue plates were kept on an orbital shaker (VEVOR® Adjustable Variable Speed Oscillator Orbital Rotator Shaker; VEVOR, Industry, CA, USA) at 20 rpm. Antigen retrieval was achieved by incubating sections in a solution of 0.1 M citric acid and 0.1 M sodium citrate (pH 6.0), first at 65°C for 5 min, then at 95°C for 10 min (Yamashita & Katsumata, 2017). Sections were allowed to cool to room temperature for 30 min, followed by three 5‐min washes in TBS. Endogenous peroxidase activity was blocked with a 3% hydrogen peroxide solution in TBS for 30 min, followed by another three 5‐min TBS washes. To reduce non‐specific binding, sections were incubated for 1 h in a blocking solution containing 1% BSA and 0.4% Triton X‐100 in TBS. The sections were then incubated overnight at 4°C with the primary antibody for FosB (cat. no. 2251, Cell Signaling Technology, Danvers, MA, USA) at a dilution of 1:2000 in the blocking solution.

On the following day, the sections were brought to room temperature for 1 h, washed three times in TBS (5 min each), and then incubated with a biotinylated secondary antibody (goat anti‐rabbit IgG, 1:400 dilution in blocking solution, Vector Laboratories, Newark, CA, USA) for 3 h at room temperature. Next, sections were treated with the Vectastain Elite avidin‐biotin complex (ABC) for 90 min (Ansorg et al., 2015). After another series of TBS washes (three 5‐min washes), the sections were developed using the nickel chloride‐enhanced diaminobenzidine (DAB) peroxidase reaction (SigmaFast™ DAB with metal enhancer, Sigma‐Aldrich, St Louis, MO, USA) for 4 min to reveal the biotinylated antibody.

**FIGURE 3:**
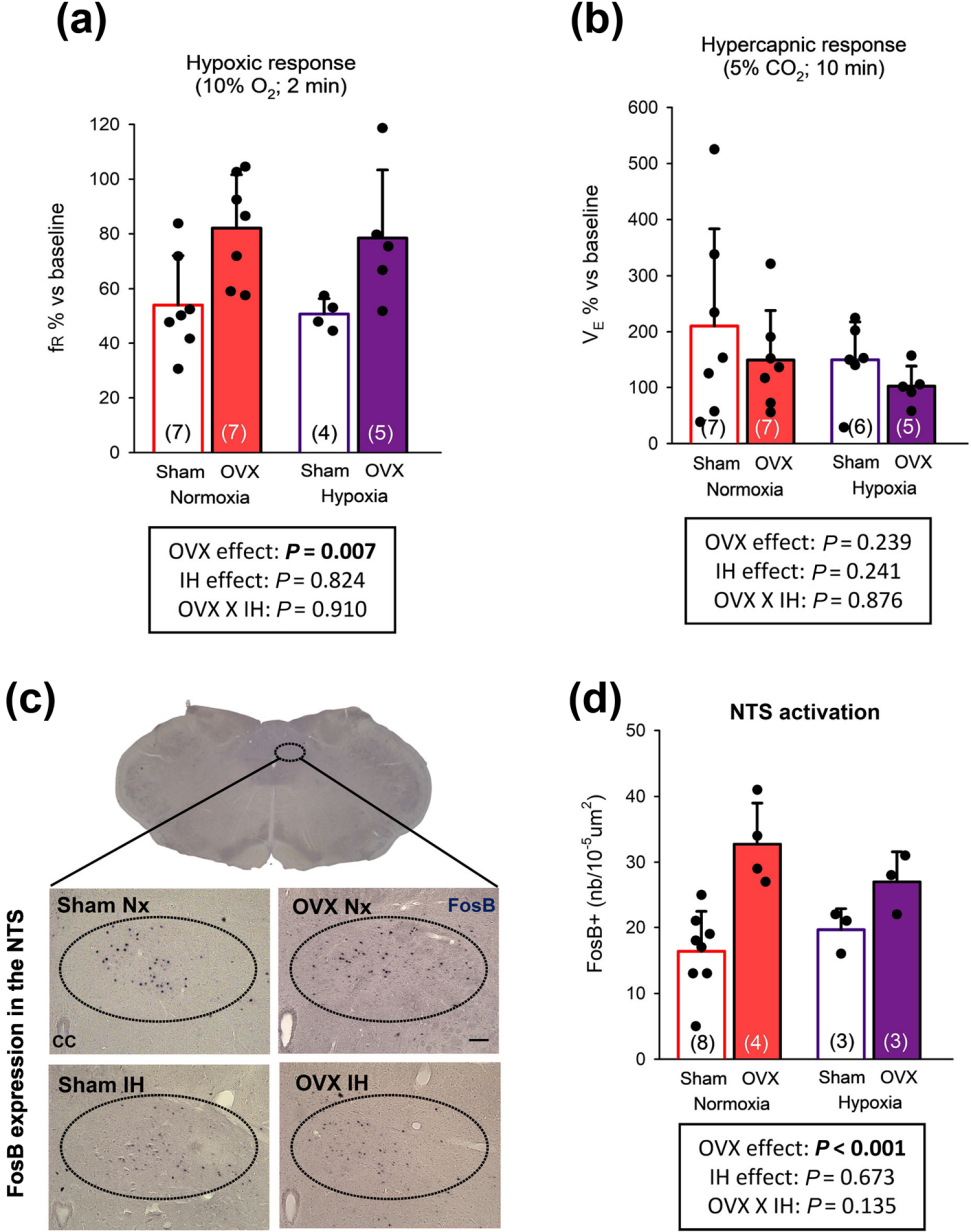
Ovariectomy (OVX), but not moderate intermittent hypoxia (IH), enhances the hypoxic ventilatory response and neuronal activation in the nucleus tractus solitarius (NTS). (a) Breathing frequency (*f*_R_) response to acute hypoxia (10% O_2_; 140 s), expressed as the percentage change from baseline 2 min after the onset of exposure, when the $F_{iO_{2}}$ reached its nadir. (b) Minute ventilation (${\dot{V}}_{E}$) response to hypercapnia (5% CO_2_, 10 min). (c) Photomicrographs comparing FosB immunolabelling in the NTS (bregma: −13.68 to −14.30 mm) from sham‐operated and ovariectomized (OVX) rats exposed to normoxia (Nx) or intermittent hypoxia (IH). CC, central canal. Scale bar = 100 µm. (d) Bar graph and individual data reporting the number of FosB expressing perikarya in the NTS. Sham‐operated rats are represented by open bars and OVX rats by filled bars; normoxia is shown in red and intermittent hypoxia in purple. Each data point represents one histological section from one animal, and group sample size (*n*) corresponds to the number of animals. Each rat is represented by an individual data point; bar height indicates group means ± SD. Group sample size (*n*) is indicated in parentheses for each group, and exact Bonferroni‐adjusted *post hoc P*‐values are shown directly on the figure when applicable.

After a final series of TBS washes (three 5‐min washes), the sections were mounted onto Fisherbrand™ Tissue Path Superfrost™ Plus Gold slides (Thermo Fisher Scientific, Waltham, MA, USA), dried for 48 h, and coverslipped with a permanent mounting medium, for subsequent microscopic examination. Slides with the FosB immunolabelled tissue were visualized under a microscope (Eclipse E600, Nikon, Tokyo, Japan) equipped with a camera (Infinity 3, Lumenera Corporation, Ottawa, ON, Canada) and stored as digital images. Images used for data analysis were captured with a ×10 magnification objective. Images were digitally acquired into ImageJ (RRID: SCR_003070). All images were filtered and adjusted equally for contrast and light levels for clarity. Analyses were performed in the PVN (bregma: −1.80 to −1.88 mm) and the caudal region of the NTS (bregma: −13.68 to 14.30 mm) based on illustrations from the rat brain stereotaxic atlas (Paxinos & Watson, 1998). The analyses were conducted in a randomized order, and the experimenters were blinded to the identities of the treatments. For each section, the right and left sides were quantified and the results averaged. The number of immuno‐positive cells was expressed as a function of the size of the structure (cell density) using standardized templates that were built according to the specific form of the structure. Figures 3c and 5a show schematic representations of the templates and representative photomicrographs.

### 2.4 Statistical analysis

**FIGURE A1:**
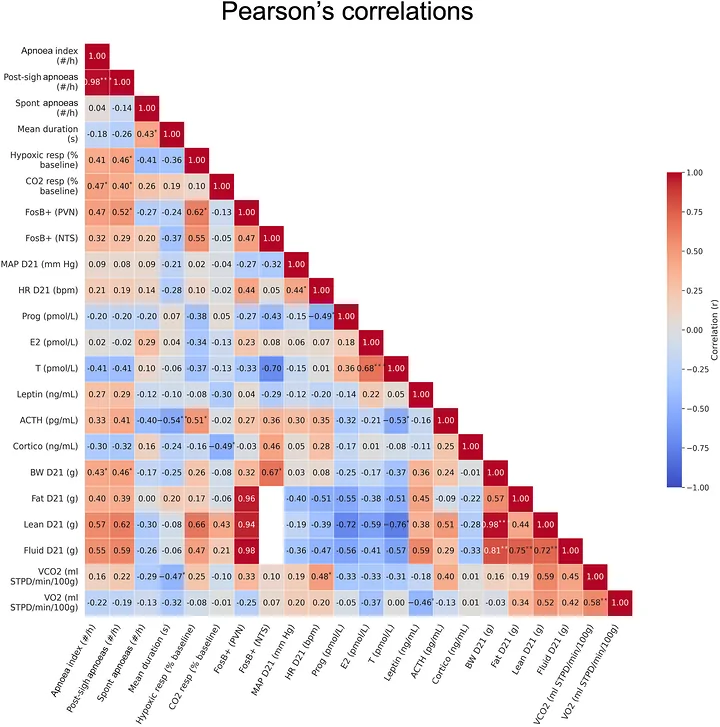
Correlation matrix of selected physiological and neuroendocrine variables. The scale of the right provides a colour indicator to visually represent the strength of the *r* values measure using Pearson's correlations. Significant correlations at * *P* < 0.05, ** *P* < 0.01 and *** *P* < 0.001.

Female rats were allocated to four experimental groups: sham normoxia (*n* = 13), sham IH (*n* = 6), OVX normoxia (*n* = 8), and OVX IH (*n* = 6). The individual rat was considered the experimental unit for all outcomes, except for food intake, which was measured per cage. Data are presented as means (SD), unless otherwise stated. Individual data points are shown in figures in accordance with the journal's statistical reporting policy. Statistical analyses were conducted using JASP (Version 0.17.1; University of Amsterdam, Netherlands). The effects of ovarian status (sham vs. OVX) and exposure (normoxia vs. IH) were assessed using a two‐way ANOVA. When a significant factorial effect (or interaction) was noted, a *post hoc* test with conditional comparisons was performed using Bonferroni's correction for multiple comparisons. Accordingly, when only one factor was significant (e.g., OVX), between‐group comparisons with a *post hoc* test were limited to that specific factor unless a factorial interaction was significant. A three‐way ANOVA for repeated measures was used to assess the effect of time on body weight (OVX and IH as independent variables and time as the repeated variable). Distribution of oestrous cycle phases between groups was tested with a χ^2^ test. Correlation matrix analysis was performed to evaluate potential relationships between physiological, anatomical and endocrine variables using Pearson's correlation test (Figure A1). A *P*‐value <0.05 was considered significant. Results of the statistical analyses can be found in the figures. Figures were created using SigmaPlot 14.5 (Grafiti LLC, Palo Alto, CA, USA) and BioRender. The visual presentation of Figure A1 was improved (e.g., abbreviations, colours, and order of presentation) using Microsoft Copilot (M365 Copilot based on the GPT‐5 chat model).

## 3 RESULTS

### 3.1 Validation of OVX and effects of IH on sex‐steroid profile and oestrous cycle

OVX reduced circulating progesterone and testosterone (Figure 1b, d); OVX did not affect oestradiol significantly (Figure 1c). Despite suggestive trends, IH did not increase sex hormone levels; however, oestradiol levels were significantly reduced by ovariectomy only in IH‐exposed OVX animals (Figure 1b–d). Comparison of the relative distribution of the phases of oestrous cycle detected in intact (sham) females between groups revealed no significant effect of IH on the oestrous cycle (Figure 1e).

### 3.2 OVX but not moderate IH augments apnoeas and respiratory instability

Cardiorespiratory and metabolic variables measured under resting (baseline) conditions were similar between groups (Table 1). Figure 2a illustrates the two types of apnoeic events recorded in sleeping females; in the bar graphs, the dark section of the bar (top) represents spontaneous apnoeas and the lighter coloured part (bottom) indicates the number of post‐sigh apnoeas. As is typically the case in rodents (Ganouna‐Cohen et al., 2023; Laouafa et al., 2017), the majority of apnoeas were observed following a sigh (Figure 2b). OVX increased apnoea frequency, whereas IH generally reduced it (spontaneous and post‐sigh apnoeas combined; Figure 2b). Neither IH nor OVX affected the mean duration of apnoeas (Figure 2c). IH exposure decreased the frequency of sighs in OVX female rats (Figure 2d).

**TABLE 1:** Baseline cardiorespiratory and metabolic variables in sham‐operated and ovariectomized female rats under normoxic and intermittent hypoxic conditions.

	Normoxia		Intermittent hypoxia	
	Sham	Ovariectomy	Sham	Ovariectomy
Breathing frequency (*f*_R_; breaths/min)	81 ± 7 (7)	82 ± 13 (8)	89 ± 18 (6)	79 ± 15 (5)
Tidal volume (*V*_T_; mL BTPS/100 g)	0.7 ± 0.3 (7)	0.6 ± 0.2 (8)	0.7 ± 0.1 (6)	0.7 ± 0.2 (5)
Minute ventilation (${\dot{V}}_{E}$; mL BTPS/min/100 g)	56 ± 21 (7)	51 ± 11 (8)	58 ± 10 (6)	54 ± 18 (5)
O_2_ consumption (${\dot{V}}_{O_{2}}$; mL STPD/min/100 g)	1.0 ± 0.3 (7)	1.0 ± 0.6 (8)	1.3 ± 0.3 (6)	1.4 ± 0.3 (5)
CO_2_ production (${\dot{V}}_{CO_{2}}$; mL STPD/min/100 g)	1.1 ± 0.3 (7)	1.4 ± 0.2 (8)	1.3 ± 0.3 (6)	1.4 ± 0.3 (5)
Respiratory exchange ratio (${\dot{V}}_{CO_{2}}$/${\dot{V}}_{O_{2}}$)	0.9 ± 0.2 (6)	1.2 ± 0.4 (6)	1.0 ± 0.2 (6)	1.0 ± 0.3 (5)
Mean arterial pressure (mmHg)	93 ± 6 (13)	90 ± 9 (8)	91 ± 3 (6)	95 ± 9 (6)
Heart rate (beats/min)	377 ± 26 (13)	381 ± 26 (8)	366 ± 33 (6)	385 ± 18 (6)

*Note*: Animals were exposed to room air (control) or moderate intermittent hypoxia ($F_{iO_{2}}$ of 0.10–30 s, 10 cycles/hour, 8 hours/day for 7 days). Values are means ± SD, with *n* shown in parentheses.

Exposure to IH did not affect the Fr response to an acute hypoxic challenge, whereas OVX increased it in both groups (Figure 3a). Neither treatment altered the ${\dot{V}}_{E}$ response to hypercapnic stimulation (Figure 3b). OVX (but not IH) augmented the number of FosB‐positive perikaya in the NTS (Figure 3c, d). Excessive responsiveness to respiratory stimuli (high loop gain) favours respiratory instability during sleep and contributes to the pathophysiology of SA (Dempsey et al., 2010). Accordingly, the occurrence of apnoeas (especially post‐sigh) was positively correlated with the intensity of the ventilatory responses to O_2_ and CO_2_ (Appendix Figure A1).

### 3.3 The obesity‐prone phenotype of OVX female is not due to exposure to IH

**FIGURE 4:**
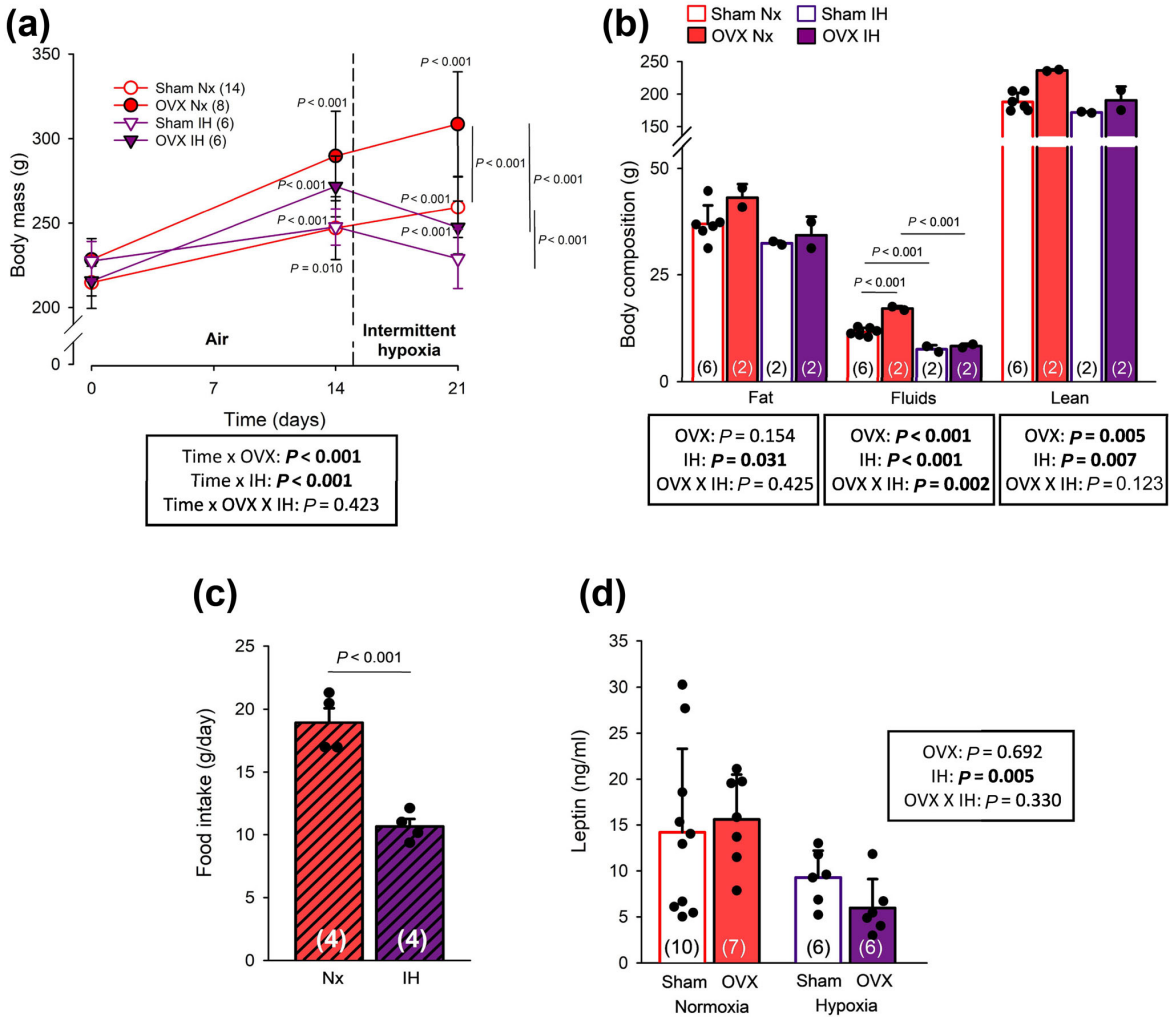
Ovariectomy (OVX), but not moderate intermittent hypoxia (IH), favours an obesity‐prone phenotype. (a) Time course of body mass progression in sham‐operated (open symbols) and ovariectomized (OVX; filled symbols) rats during the 14‐day post‐surgery recovery period in room air; the vertical dashed line indicates the onset of the exposure to normoxia or intermittent hypoxia for 7 days. Symbols and lines represent group means ± SD. (b) Endpoint body composition, showing fat mass, fluid mass and lean mass. (c) Mean daily food intake (chow) pooled according to exposure condition (normoxia; Nx vs. intermittent hypoxia; IH). (d) Plasma leptin levels measured at the end of the protocol. Sham‐operated rats are represented by open bars and OVX rats by filled bars; normoxia is shown in red and intermittent hypoxia in purple. Each rat is represented by an individual data point; bar height indicates group means ± SD. Group sample size (*n*) is indicated in parentheses for each group, and exact Bonferroni‐adjusted *post hoc P*‐values are shown directly on the figure when applicable.

Prior to the onset of the protocol (day 0), body weights were similar between groups (Figure 4a). All animals gained weight and the larger increase in OVX females was significant at day 14. Subsequent exposure to IH (day 15) induced weight loss, regardless of ovarian status whereas air‐exposed females continued to gain weight (Figure 4a). Technical issues with the NMR apparatus prevented us from assessing body composition in all animals; however, sufficient data were gathered to suggest that, by comparison with females maintained in normoxia, IH reduced body fat (Figure 4b). Intermittent hypoxia reduced the amount of body fluids whereas OVX increased it; this effect was most notable in air‐exposed females (Figure 4b). Ovariectomy augmented lean mass whereas IH reduced it (Figure 4b). These results are consistent with the lower food intake observed in IH‐ versus air‐exposed rats (Figure 4c); importantly, sham and OVX females were co‐housed (one sham and one OVX per cage; 2 animals per cage). Exposure to 1 week of moderate IH decreased plasma leptin levels (Figure 4d). O_2_ consumption (${\dot{V}}_{O_{2}}$), CO_2_ production (${\dot{V}}_{CO_{2}}$) and respiratory exchange ratio (${\dot{V}}_{CO_{2}}$/${\dot{V}}_{O_{2}}$) were measured as indicators of metabolism (Table 1); none were affected by experimental treatment and showed only limited correlations with the main physiological outcomes or hormones (Figure A1). Obesity is an important risk factor for SA (Dempsey et al., 2010); in line with this principle, the body weight measured at the end of the protocol was positively correlated with post‐sigh apnoea frequency (Figure A1).

### 3.4 OVX (but not IH) activates the stress pathways

**FIGURE 5:**
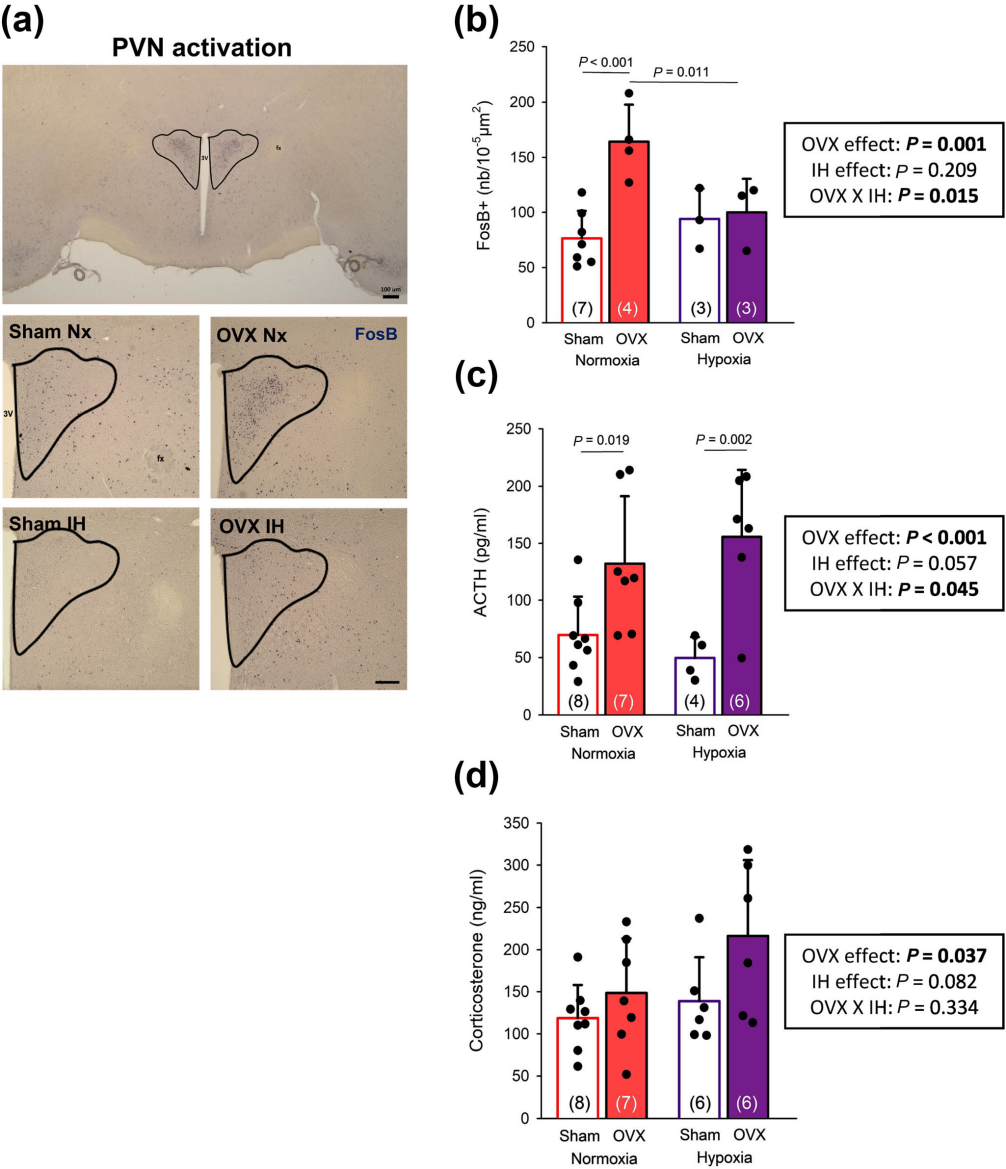
Ovariectomy (OVX), but not moderate intermittent hypoxia (IH), activates the hypothalamo–pituitary–adrenal axis in adult female rats. (a) Photomicrographs comparing FosB immunolabeling in the paraventricular nucleus of the hypothalamus (PVN; bregma −1.80 mm) in sham‐operated and ovariectomized (OVX) rats exposed to normoxia (Nx) or intermittent hypoxia (IH). 3V, third ventricle. Scale bar: 100 µm. (b–d) Bar graphs and individual data reporting the number of FosB‐expressing perikarya in the PVN (b), where each data point represents one histological section from one animal, and group sample size (*n*) corresponds to the number of animals; plasma adrenocorticotropic hormone (ACTH) (c); and corticosterone (d). Sham‐operated rats are represented by open bars and OVX rats by filled bars; normoxia is shown in red and intermittent hypoxia in purple. Each rat is represented by an individual data point; bar height indicates group means ± SD. Group sample size (*n*) is indicated in parentheses for each group, and exact Bonferroni‐adjusted *post hoc P*‐values are shown directly on the figure when applicable.

The ‘resting’ activation level of the HPA axis was first assessed by quantifying the number of FosB‐positive perikarya in the PVN. The photomicrograph presented in Figure 5a illustrates the FosB immunolabeling signal typically obtained in our experiments. Population data show that OVX augmented the number of FosB‐expressing cells; this effect was most notable in normoxia‐exposed females (Figure 5b). Ovariectomy also increased plasma ACTH levels in normoxia and IH‐exposed females (Figure 5c); OVX also augmented plasma corticosterone levels (Figure 5d).

The PVN projects to numerous medullary regions regulating reflexive responses to respiratory stimuli (Ruyle et al., 2023; Tenorio‐Lopes & Kinkead, 2021). Here, PVN labelling density and stress hormones levels were all positively correlated with the magnitude of the hypoxic ventilatory response (Figure A1). PVN labelling density was also correlated with post‐sigh apnoea frequency (Figure A1).

## 4 DISCUSSION

While sex‐based differences in sleep apnoea (SA) are well recognized, elucidating the reasons why SA and related co‐morbidities only manifests in a subset of postmenopausal women remains a key challenge. To address this knowledge gap, we used an integrative approach characterizing cardiorespiratory, neuroendocrine and metabolic readouts after ovariectomy (OVX) with and without exposure to a moderate intermittent hypoxia (IH) paradigm. We show that OVX alone has significant impacts on respiratory, endocrine and metabolic functions. Contrary to our hypothesis, 1‐week exposure to moderate IH alone had limited effects on these outcomes and did not exacerbate the consequences resulting from OVX in adult female rats. Rather, the effects of OVX and IH were outcome‐specific: OVX increased apnoea frequency and activated the HPA axis, whereas IH generally reduced apnoea frequency and affected metabolic variables. While our data support the importance of body weight as a risk factor for SA‐related respiratory instability, the associations between stress‐pathway markers and respiratory disturbances bring support to the notion that stress is a driving force in the onset of SA (Kinkead et al., 2021).

### 4.1 Hormonal profile following IH and OVX

Compared with castration in males (Ganouna‐Cohen et al., 2023), the drop in sex steroids following ovariectomy is more limited in females (Tenorio‐Lopes et al., 2020). In the present study, the sex steroid levels are comparable to those observed in 1‐year‐old female rats (Ambrozio‐Marques et al., 2026; Fournier et al., 2015). Unlike males, females compensate sex‐steroid production from extragonadal sources such as the hypothalamus, which contributes to progesterone synthesis following ovariectomy (Micevych et al., 2003) whereas oestradiol synthesis occurs via aromatization taking place in adipose tissue and the liver (Hetemäki et al., 2025). The significant correlation between oestradiol and testosterone is in line with the contribution or aromatase in this process (Figure A1).

The effects of IH on circulating gonadal steroids in intact females vary between studies (Liu et al., 2020; Marcouiller et al., 2020; Song et al., 2022). Here, IH exposure was associated with a modest upward shift in circulating progesterone, oestradiol and testosterone in sham‐operated rats, whereas this pattern was absent after OVX, consistent with a gonad‐dependent hormone release mediated by sympathetic innervation of the ovaries (Astudillo‐Guerrero et al., 2024), sympathetic innervation of testes (Gerendai et al., 2005) and/or direct hypoxic stimulation of Leydig cell steroidogenesis (Hwang et al., 2009). These results suggest (albeit indirectly) that our protocol activated the sympathetic pathway.

### 4.2 OVX exerts greater impact on respiratory function than IH

The higher apnoea frequency observed in OVX females is in line with other rodent studies (Laouafa et al., 2017); however, the apnoea values obtained in OVX‐IH rats were unexpected as they contrast with the significant rise of apnoea frequency observed in other OVX rats subjected to the same IH protocol (Joseph et al., 2020; Laouafa et al., 2017). Considering that rats were from the same strain, of a similar age and subjected to the same IH protocol, the major differences between the two studies are that our animals were born and raised in our animal care facilities, and housed in pairs rather than in isolation in the previous study (Laouafa et al., 2017). Housing conditions during the juvenile period affect respiratory responses (Fournier et al., 2011, 2012), prolonged single housing can be stressful (Mumtaz et al., 2018), and obtaining rats from a vendor exposes animals to transportation stress. This implies that once they reach adulthood, animals born and raised ‘in house’ are less responsive to stress such that a more severe IH may be necessary to significantly disrupt the respiratory control network. Although the reduction in food intake and body weight indicates that the IH protocol was physiologically active, IH did not increase stress hormones or FosB labelling in the PVN, supporting the interpretation that moderate IH did not elicit detectable HPA‐axis activation under these conditions. As we discuss below, the positive relationship between FosB‐expressing cells in the PVN and the occurrence of post‐sigh apnoeas highlights the contribution of stress to SA pathophysiology.

Excessive responsiveness to respiratory stimuli (high loop gain) is a key mechanism in the pathophysiology of SA as it promotes respiratory instability and airway collapse during sleep (Deacon & Catcheside, 2015; White & Younes, 2012). The positive correlations between reflexive responses to O_2_ and CO_2_ challenges and apnoeic events reported here are in line with this principle. However, while OVX increased the acute hypoxic ventilatory response in both normoxia and IH exposed rats, IH exposure did not affect the ventilatory responses to either stimulus. Metabolic disturbance leading to obesity is another important risk factor for SA and a positive correlation between body weight and the apnoea index suggests that the weight loss resulting from IH exposure may have limited the effect of the augmented hypoxic response on apnoea frequency. Mechanistically, this implies that the chemoreflex increase observed here is not sufficient to augment apnoeas in sleeping females; other factors (e.g., weight gain, stress) may be necessary.

The hypercapnic response is another determinant of respiratory stability during sleep. The magnitude of the responses reported here were within the normal range (Marques et al., 2015), but neither treatment affected this chemoreflex. Our data do not allow us to explain why OVX selectively affected the O_2_‐ but not CO_2_‐induced hyperventilatory responses; however, considering the OVX‐related increase in responsiveness to O_2_, the rise in arterial O_2_ resulting from CO_2_‐induced hyperpnoea may counteract potential treatment‐related increases. The NTS receives chemosensory signals from CO_2_ and O_2_ sensing structures (retrotrapezoid nucleus and carotid bodies, respectively), but unfortunately, the changes in FosB expression observed in the NTS do not inform us on that point because the FosB expression dynamics are too slow to reflect changes related to acute exposure to respiratory stimuli. Thus, the increase in FosB expressing cells in NTS following OVX (but not IH) more likely reflects augmented tonic inputs. In addition to chemosensory afferents, the NTS receives inputs from multiple sources, including the PVN (Ruyle et al., 2023). The sum of these inputs activates central autonomic pathways, triggering hyperventilation, sympathetic activation and, with time, recurrent NTS activation linked to sleep fragmentation and hypertension (Chopra et al., 2016; Laouafa et al., 2017, 2018).

### 4.3 Cardiovascular function is unaffected by OVX or IH

Hypertension is an important co‐morbidity of SA. While its prevalence and consequences are more severe in men, the risk of cardiovascular diseases and related complication increases with age in women (Drury et al., 2024). The progressive loss of the protective actions of ovarian hormones is often invoked to explain the rise of cardiovascular disease following menopause, but the addition of other risk factors is necessary to exacerbate this physiological change. Activation of stress‐related mechanisms is of interest here as it contributes to many established factors including sympathetic activation and obesity (Reckelhoff & Fortepiani, 2004). In line with this principle, data show that OVX alone does not affect mean arterial pressure (MAP; Laouafa et al., 2017; Appiah et al., 2025; Table 1), but unlike other studies using similar protocols, IH failed to augment MAP or heart rate. This is not a complete surprise considering that the MAP increase following moderate IH are usually modest (5–10 mmHg). But in addition to differences associated with housing and transport evoked previously, differences in technique (tail cuff vs. telemetry, approach to data analysis) and the time when cardiovascular data were obtained (inactive vs. active phase) may also contribute to these differences (Appiah et al., 2025). Moreover, our experiment's timing (2 vs. 1‐week post‐OVX) might have missed the peak OVX effect.

While the changes in body weight, metabolism and leptin confirm the efficacy of our IH protocol, the lack of effect on blood pressure and PVN activation indicate that a slightly more severe protocol (3 min vs. 30 s bouts of 10% O_2_) may be necessary to reveal OVX‐ and IH‐related cardiovascular disturbances (Appiah et al., 2025). It remains possible that a deeper nadir (e.g., 6–9%) would have produced stronger cardiovascular and neuroendocrine effects. However, hypoxic severity alone is unlikely to fully account for these differences, as comparable nadir O_2_ levels can yield distinct cardiovascular outcomes depending on the overall structure of the protocol (Sforza & Roche, 2016). In addition, the relatively slow oxygen kinetics (21% to 10% O_2_ in 90 s, 10% for 30 s, then back to 21% in 70 s) of our housing cages used for IH exposure likely reduced the time spent at peak hypoxia, thereby attenuating the effective IH stimulus. As mentioned previously, the lower sensitivity of our ‘born‐in‐house’ animals to stressors may account for the lack of an IH effect.

### 4.4 OVX and IH have opposing effects on body weight and composition

Although obesity has a greater impact on the severity of SA in men, it remains an important risk factor in women, especially following menopause (Martins & Conde, 2021; Subramanian et al., 2012). Here, a rise in apnoeas occurred alongside OVX‐induced weight gain and increased body fluids and lean mass, a phenomenon comparable to age‐related ovarian decline (Acharya et al., 2023; Ambrozio‐Marques et al., 2025). Our data showing that apnoea frequency correlates better with body weight than body fat was unexpected but may reflect the low number of MRI measurements that could be obtained.

Conversely, drops in body weight, fat and fluids along with food intake are well‐documented in IH‐exposed animals (Moreau & Ciriello, 2013). Intermittent hypoxia generally affects energy balance via hypothalamic satiety signals such as leptin, but the effects vary greatly between studies. The leptin values measured in OVX females are consistent with established links between obesity, hyperleptinaemia and SA. In addition to its role in energy balance, leptin generally upregulates ventilatory control via actions on the carotid bodies, the NTS/hypothalamic respiratory pathways (Yao et al., 2016); thus, the higher leptin levels observed in OVX females may contribute to the augmented hypoxic response. In contrast, IH reduced leptin. As the white adipose tissue is the primary site of secretion of leptin, the IH‐related loss fat mass likely explains this effect. The appetite‐suppressive effects of IH may contribute to the loss of weight and body fat; however, this effect may only be transient as mice exposed to more chronic protocols (6–18 weeks) ultimately gain weight (Barnes et al., 2023).

### 4.5 Limitations, conclusion and perspectives

Although OVX is a highly convenient approach to mimic physiological changes associated with menopause, it does not fully recapitulate the gradual effect of ageing and the associated endocrine changes. Accordingly, age‐related factors may also contribute to the development and phenotype of sleep apnoea in women, through female‐specific changes such as an increase in upper‐airway collapsibility and reduced responsiveness of upper‐airway muscle activity, which are not captured in the present model (Popovic & White, 1995; Qiu & Mateika, 2021). While OVX disrupts respiratory control and augments respiratory instability during sleep, the physiological significance of those changes remains uncertain in our study. Blood pressure did not increase, and we do not know if the rise in apnoea index was associated with significant drops in arterial O_2_. We can nonetheless conclude that OVX, much like menopause, does not induce a disease state but represents a ‘high risk’ condition in which the stress pathways are more active. Sex hormones affect the HPA axis as oestrogen and androgen receptors are expressed in PVN neurons and their afferent sites, allowing gonadal hormone fluctuations to regulate stress reactivity (Gordon et al., 2016; Handa & Weiser, 2014). Much like OVX, menopause is linked to a rise in circulating cortisol, and menopause‐associated neuroendocrine changes, particularly HPA axis dysregulation, have been reported in various health consequences; however, its specific role in SA progression remains unclear (Cohn et al., 2023; Hantsoo et al., 2023; Salardini et al., 2025). Thus, without the protective actions of ovarian hormones, exposure to stress will likely have more severe physiological consequences (Kinkead et al., 2021). Our recent work has shown that in rats, age‐related loss of ovarian function also reveals the latent effects of experiencing stress during early life on cardiorespiratory function (Ambrozio‐Marques et al., 2026).

A central role for stress‐related HPA axis activation is an attractive hypothesis to explain the divergent ageing trajectories that women can experience as they enter menopause. We must keep in mind, however, that the present study is based mainly on observations and correlations and causality remains untested. Considering that oestrogen replacement therapy attenuates HPA‐axis responses to certain emotional stressors in postmenopausal women, testing the effects of this hormone on the respiratory network in females is a promising future research avenue.

## AUTHOR CONTRIBUTIONS

The experiments were conducted at the Research Centre of the Quebec Heart and Lung Institute, Québec City, Canada. Stéphanie Fournier, François Marcouiller, Vincent Joseph and Richard Kinkead conceptualized and designed this study. Marianne Gagnon, Stéphanie Fournier and François Marcouiller performed the experiments and analysed the data. Natalie J. Michael and Vincent Joseph contributed to the interpretation of the data. Marianne Gagnon and Richard Kinkead wrote the paper. All authors critically reviewed and edited the manuscript, approved the final version of the manuscript, and agree to be accountable for all aspects of the work. All persons designated as authors qualify for authorship, and all those who qualify for authorship are listed.

## CONFLICT OF INTEREST

None declared.

## GENERATIVE AI STATEMENT

Microsoft Copilot (M365 Copilot based on the GPT‐5 chat model, Microsoft) was used to assist with the visual presentation of Figure A1, including optimization of font size, abbreviation formatting and overall figure organization. The tool was used solely to improve the clarity and presentation of the figure. Generative AI tools were not used for data collection, data analysis, statistical analysis, or interpretation of results. All AI‐assisted output was reviewed, edited and verified by the authors, who take full responsibility for the content of the manuscript.

## Data Availability

All data supporting the results are reported in the manuscript. Raw results can be provided upon request.
